# Reliability of questionnaire The International Fitness Scale: a systematic review and meta-analysis

**DOI:** 10.31744/einstein_journal/2020RW5232

**Published:** 2020-07-23

**Authors:** Débora de Almeida Pereira, Jânio Luiz Correia, Nelson Carvas, Ricardo de Freitas-Dias

**Affiliations:** 1 Graduate Program in Adolescent Medicine – Determinants of Health in Adolescents Universidade de Pernambuco CamaragibePE Brazil Graduate Program in Adolescent Medicine – Determinants of Health in Adolescents, Universidade de Pernambuco, Camaragibe, PE, Brazil.; 2 Department of Physical Therapy Universidade Pernambuco PetrolinaPE Brazil Department of Physical Therapy, Universidade Pernambuco, Petrolina, PE, Brazil.; 3 Universidade Ibirapuera São PauloSP Brazil Universidade Ibirapuera, São Paulo, SP, Brazil.

**Keywords:** The International Fitness Scale, IFIS, Physical fitness, Muscle strength, Cardiorespiratory fitness, Self report

## Abstract

**Objective:**

To perform a systematic literature review and meta-analysis to investigate the reliability of The International Fitness Scale questionnaire for assessing overall physical fitness and related components.

**Methods:**

PubMed^®^, BIREME, SciELO, EMBASE, SPORTDiscus, LILACS and Cochrane databases were searched using the following search terms: “The International Fitness Scale”, “International Fitness Scale” and “IFIS”. Article selection and data extraction were performed according to the following eligibility criteria: reliability and/or validity study of the measure tools of The International Fitness Scale; adoption of the The International Fitness Scale as a reference criterion (gold standard) and being an original article. Quality of the study was considered based on Assessment of Reliability Studies. Data analysis used Kappa coefficient of agreement, Cochran and the Higgins I^2^ test. Sensitivity analysis was conducted using the withdrawal model.

**Results:**

A total of seven articles were included in the analysis. Test-retest reliability coefficients ranged from 0.40 to 0.99, with most studies achieving values ≥0.60, indicative of moderate to substantial reliability.

**Conclusion:**

In spite of appropriate test-retest scores attributed to most reliability indicators, heterogeneity among the studies remained high. Therefore, further studies with low risk of bias are needed to support the reliability of the self-reported The International Fitness Scale.

## INTRODUCTION

Physical fitness is a predictor of health problems. Satisfactory fitness levels contribute to health problem prevention and functional capacity maintenance and improvement, and limit the development of chronic degenerative dysfunctions, leading to better quality of life.^( 1 )^

Direct physical fitness measurement methods are considered gold standard. However, these methods have limitations, such as need for laboratories, high costs of equipment, need for a specialized team and difficult interpretation of findings.^( 2 , 3 )^ Questionnaires are therefore an alternative for epidemiological studies, particularly in developing countries,^( 4 )^ due to their user-friendly nature, low cost, reliability and reproducibility.^( 5 )^

Multicenter research investigating adolescent lifestyle in Europa has led to the development of the International Fitness Scale (IFIS) self-reported questionnaire for assessing overall physical fitness and related components (cardiorespiratory fitness, muscle strength, speed/agility and flexibility).^( 2 )^ This questionnaire was originally validated in the English language for adolescents aged 12 to 17 years,^( 2 )^ then adapted and translated into nine languages (German, Austrian German, Greek, Flemish, French, Hungarian, Italian, Spanish and Swedish)^( 2 )^ and validated for use in different populations (male and female children, youngsters and adults).^( 3 , 6 - 8 )^ Results derived from IFIS revealed associations with risk factors for cardiovascular diseases and metabolic syndrome.^( 3 , 6 , 8 )^

The IFIS has been employed in several international research studies. Still, instruments with accurate psychometric properties, capable of reproducing a given outcome consistently within time and space, or across different observers (reliability), are required for studies aimed to estimate physical fitness, identify associated risk factors, analyze relations with different outcomes, and assess effectiveness of training programs.^( 9 )^

Given the significance of physical fitness measurement using reliable, user-friendly instruments, and the growing interest in this field, this study set out to conduct a systematic review and meta-analysis of the available literature, in order to determine whether IFIS is a reliable tool for assessing overall physical fitness and related components.

## METHODS

### Protocol and registration

This systematic review was conducted in compliance with Reporting Items for Systematic Reviews and Meta-Analyses (PRISMA) recommendations. The review protocol was registered in the International Prospective Register of Systematic Reviews (PROSPERO), under no. CRD42018117472.

### Search strategy

Literature search included articles published up to September 2019 and listed in the following data bases: MEDLINE via PubMed^®^, BIREME, Scientific Electronic Library Online (SciELO), EMBASE, SPORTDiscu *s* , LILACS and Cochrane Central, regardless of type of study, population, language, participant age and sex, and publication date. Studies were searched using the following descriptors *: “Physical Fitness” and “Self-report”* (controlled) *and* “ *The International Fitness Scale* ”; “ *International Fitness Scale* ”; “IFIS” (non-controlled). Terms were combined using the Boolean operator (OR). The [TIAB] field code was used to limit exhibition to articles containing selected terms in the title and abstract ( Table 1 ).

**Table 1:** Search strategy

Data bases PubMed^®^, BIREME, SciELO, EMBASE, SPORTDiscus, LILACS and Cochrane: *The International Fitness Scale* [TIAB] *OR* [TIAB] *International Fitness Scale* [TIAB] *OR* IFIS [TIAB]
EMBASE (interface does not allow use of the [TIAB] field code): *The International Fitness Scale OR International Fitness Scale* [TIAB] *OR* IFIS.

### Study selection

An assessment form developed based on inclusion and exclusion criteria and calibrated prior to screening was used for study selection. Inclusion criteria were as follows: studies addressing reliability and/or validity of the IFIS measurement instrument; original research articles involving human beings; publication in journals indexed in the selected databases. Review articles were excluded. The Mendeley Reference Manager Software (https://www.mendeley.com/) was used to ensure independent selection and assessment across reviewers.

Duplicate studies were excluded. Two blinded, independent reviewers selected studies in two steps: title and abstract screening and full text reading. In the first step, titles and abstracts were examined according to predefined eligibility criteria for identification of relevant studies. Studies selected by at least one reviewer were included in the subsequent step. These were then read in full and examined by reviewers based on eligibility criteria, using an evaluation form.

Articles selected for full text reading were submitted to cross-reference search for identification of relevant studies that might not have come up in electronic search.

### Data extraction

Data extraction was performed according to the Cochrane Handbook for Systematic Reviews of Interventions.^( 10 )^ Data extracted from studies satisfying eligibility criteria were entered into an electronic Excel spreadsheet (Microsoft Excel software *;* Microsoft Corporation, WA, USA). The following pieces of data were extracted: first author, title and year of publication; type of study; descriptive (overall sample size, sample size per sex, age group and country where the study was conducted, and sampling procedures) and reliability (Kappa values and 95%CI) data.

Two independent raters extracted descriptive and outcome data from selected articles. The GRADE System was used to examine overall quality of evidence.^( 11 )^ Unresolved discrepancies between raters were examined by a third rater. Prior to data extraction, raters received training in calibration to ensure inter-rater consistency and data extraction spreadsheet refinement.

### Methodological quality assessment: risk of bias

Methodological quality of selected studies was assessed using the Quality Appraisal of Reliability Studies (QAREL). This instrument includes 11 items in the following domains: items 1 and 2 – sampling bias, participants and rater representativeness; items 3 to 7 – blinding of raters; item 8 – variations in order of examination; item 9 – appropriate time intervals between repeated measures; item 10 – correct test application and interpretation; item 11 – appropriate statistical analysis. Items may be answered with “yes”, “no”, “unclear” or “not applicable” (items 3, 4, 5, 6 and 8); “yes” and “no” suggest good and poor study quality, respectively.^( 12 )^

Inconsistencies in this study were discussed among authors and a final decision reached by consensus, according to Cochrane Handbook for Systematic Reviews recommendations.^( 10 )^ In the absence of consensus, a third author was consulted, reasons for article exclusion examined, and a decision made.

### Data analysis

Reliability was tested using the Kappa coefficient of agreement; sample size was used for grouped Kappa calculation. The random effects model was chosen over the fixed effects model due to varying levels of physical fitness among individuals, which may have reflected the impacts of physical activity during childhood and adolescence on adult life.^( 13 )^ Kappa coefficients of agreement were interpreted as follows: none <0.00; slight, 0.00 to 0.20; fair, 0.21 to 0.40; moderate, 0.41 to 0.60; substantial, 0.61 to 0.80; almost perfect, 0.81 to 1.00.^( 14 )^

Statistical heterogeneity was investigated using the Cochran Q test (level of significance, p<0.10). Statistical inconsistency was investigated using the Higgins I^2^test,^( 15 )^ as follows: ≤40%, low heterogeneity; 30% to 60%, moderate heterogeneity; >50% to 90%, substantial heterogeneity; and >75% to 100%, considerable heterogeneity.^( 10 )^ Whenever I^2^ >50% and tau squared (𝛕^2^) >1, in the presence of statistical significance (p<0.10), heterogeneity was rated significant and reasons investigated. Statistical analyses were performed using software (R package meta; R 3.5.1).

### Sensitivity analysis

Subgroup analysis was conducted to explain study heterogeneity. Effects were divided by study population and sampling bias, then meta-regression calculation performed.

## RESULTS

**Figure 1:**
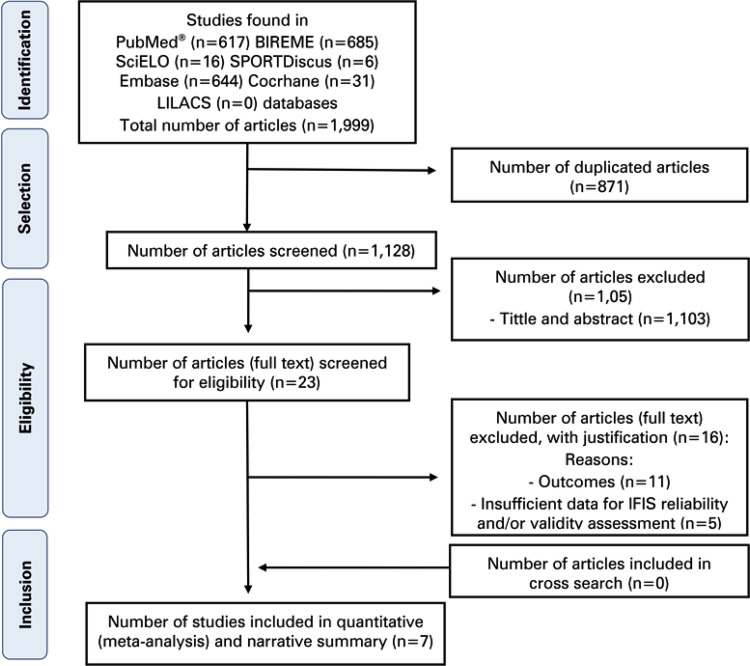
Study selection flowchart IFIS: The International Fitness Scale.

A total of 1,999 articles were found in the selected databases. Of these, 871 (duplicates) were excluded. Title/abstract screening and full text reading included 1,128 and 23 articles respectively, with 99.2% agreement between raters. Seven of these articles satisfied eligibility criteria and were included in the quantitative narrative analysis of this meta-analysis ( Figure 1 ).

### Study characteristics

Narrative and quantitative summary in this meta-analysis comprised seven studies.^( 2 , 3 , 6 , 7 , 8 , 16 , 17 )^ Selected articles were published between 2011 and 2019. Sample size ranged from 89 to 413. Overall, five studies^( 2 , 3 , 6 , 8 , 16 )^ included participants of both sexes; male sex prevailed in three studies,^( 3 , 8 , 16 )^ one study was based exclusively on women^( 7 )^ and one study did not describe sex distribution of the sample.^( 17 )^ The recruitment process consisted primarily of random sampling,^( 2 , 6 , 7 , 8 , 16 )^with two studies involving convenience sampling.^( 3 , 17 )^ Mean participant age varied widely among studies, ranging from 3 to 65 years. This sample included five observational test-retest reliability studies,^( 2 , 3 , 6 , 16 , 17 )^ one cluster randomized trial,^( 8 )^and one cross-sectional study^( 7 )^( Table 2 ).

**Table 2:** Summary and characteristics of findings of studies investigating reliability of The International Fitness Scale instrument for physical fitness assessment

Author	Type of study	Sample size	Characteristics of participants	Interval between applications	IFIS Application
Ortega et al.^(2)^	Observational, test-retest reliability study	n=277	Sex: female (51%) and male (49%). Age group: 12.5 to 17.5 years. Countries: Grece, Germany, Belgium, France, Hungary, Austria, Italy, Sweden and Spain. Health status: free from acute infection of any kind or long standing disease <1 week prior to inclusion in the study	2 weeks	Not reported
Ramírez-Velez et al.^(3)^	Observational, test-retest reliability study	n=229	Sex: female (45.85%) and male (54.15%). Age group: 9 to 17.9 years. Country: Colombia. Health status: no clinical diagnosis of cardiovascular disease and/or type 1 or 2 *diabetes mellitus* , not pregnant, no consumption of alchool or other drugs	1 week	Not reported
Ortega et al.^(6)^	Observational, test-retest reliability study	n=181	Sex: female (73.48%) and male (27.52%). Age group: 18 to 43 years. Country: Spain	2 weeks	Not reported
Álvarez-Gallardo et al.^(7)^	Cross-sectional study	n=413	Sex: female. Age group: 37 to 65 years. Country: Spain. Health status: affected with fibromyalgia	1 week	Not reported
Sánchez-López et al.^(8)^	Cluster randomized trial	n=245	Sex: female (54%) and male (46%). Age group: 9 to 12 years. Country: Spain	2 weeks	Not reported
Olivares et al.^(16)^	Observational, test-retest study	n=89	Sex: female (37.8%) and male (62.2%). Age group: 12 to 18 years. Country: Chile	2 weeks	During physical education class Examiners graduated in Education in Physical Education and previously trained
De Moraes et al.^(17)^	Observational, test-retest reliability study	n=190 children and n=110 adolescents	Sex distribution of adolescent participants not reported by authors. Children aged 3 to 10 years (mean 6.7±2.1 years) and adolescents aged 11 to 17 years (14.6±1.8 years). Country: Brazil	15 days	Data collected over the course of 5 visits: (1) Project explanation and ICF handed out to parents; (2) Handing out of self-report questionnaire; (3) collection of filled out questionnaire; (4) second application; (5) collection of filled out questionnaire

IFIS: International Fitness Scale; ICF: Informed Consent Form.

Studies in this sample reported test-retest reliability estimates based on Kappa agreement coefficients. Time intervals between examinations ranged from 1 to 2 weeks, with 2-week intervals used in most studies^( 2 , 6 , 8 , 16 , 17 )^ and 1-week intervals limited to two studies.^( 3 , 7 )^

### Risk of bias

Inter-rater agreement regarding risk of bias was 94.8% (4 inconsistencies across 77 items examined). Overall, study participants^( 2 , 3 , 6 , 7 , 8 , 16 , 17 )^ were representative of those to whom the authors intended the results to be applied (QAREL item Q2) and intervals between repeated measurements of the target variable (QAREL item Q9) were reported.

As regards primary sources of bias, blinding of raters to findings of other raters or to their own previous findings, to results of the reference standard accepted for the target variable, to clinical information, to additional cues and to order of examination was not reported in any of the studies. In two studies,^( 2 , 6 )^ tests were conducted by raters who were representative of those to whom the authors intended the results to be applied. Finally, correct test application and appropriate interpretation, as well as appropriate statistical analysis, were performed in studies in this sample ( Table 3 ).

**Table 3:** Methodological quality assessment according to Quality Assessment of Reliability Studies checklist

Study	Q1	Q2	Q3	Q4	Q5	Q6	Q7	Q8	Q9	Q10	Q11
Ortega et al.^(2)^	Y	Y	NC	NC	NC	NC	NC	NC	Y	Y	Y
Ramírez-Vélez et al.^(3)^	Y	NC	NC	NC	NC	NC	NC	NC	Y	Y	Y
Ortega et al.^(6)^	Y	NC	NC	NC	NC	NC	NC	NC	Y	Y	Y
Álvarez-Gallardo et al.^(7)^	Y	NC	NC	NC	NC	NC	NC	NC	Y	Y	Y
Sánchez-López et al.^(8)^	Y	Y	NC	NC	NC	NC	NC	NC	Y	Y	Y
Olivares et al.^(16)^	Y	NC	NC	NC	NC	NC	NC	NC	Y	Y	Y
De Moraes et al.^(17^	Y	NC	NC	NC	NC	NC	NC	NC	Y	Y	Y

Q1: Was the test evaluated in a sample of subjects who were representative of those to whom the authors intended the results to be applied?; Q2: Was the test performed in a sample of subjects who were representative of those to whom the authors intended the results to be applied?; Q3: Were raters blinded to the findings of other raters during the study?; Q4: Were raters blinded to their own prior findings of the test under evaluation?; Q5: Were raters blinded to the results of the reference standard for the target variable being evaluated?; Q6: Were raters blinded to clinical information that was not intended to be provided as part of the testing procedure or study design?; Q7: Were raters blinded to additional cues that were not part of the test?; Q8: Was the order of examination varied?; Q9: Was the time interval between repeated measurements compatible with the stability (or theoretical stability) of the variable being measured?; Q10: Was the test applied correctly and interpreted appropriately?; Q11: Were appropriate statistical measures used?; Y: yes; NC: not clear.

### Summary of reliability findings

According to Kappa coefficients, overall test-retest reliability ranged from 0.73 to 0.81 (substantial to almost perfect agreement). When all items assessed in selected studies were accounted for, reliability ranged from 0.40 to 0.99 (fair to almost perfect), with more than 50% (26 out of 40 items) achieving values ≥0.60 or moderate to substantial level of reliability - and 30% (12 out of 40 items) achieving almost perfect reliability as per Landis et al.^( 14 )^

**Figure 2:**
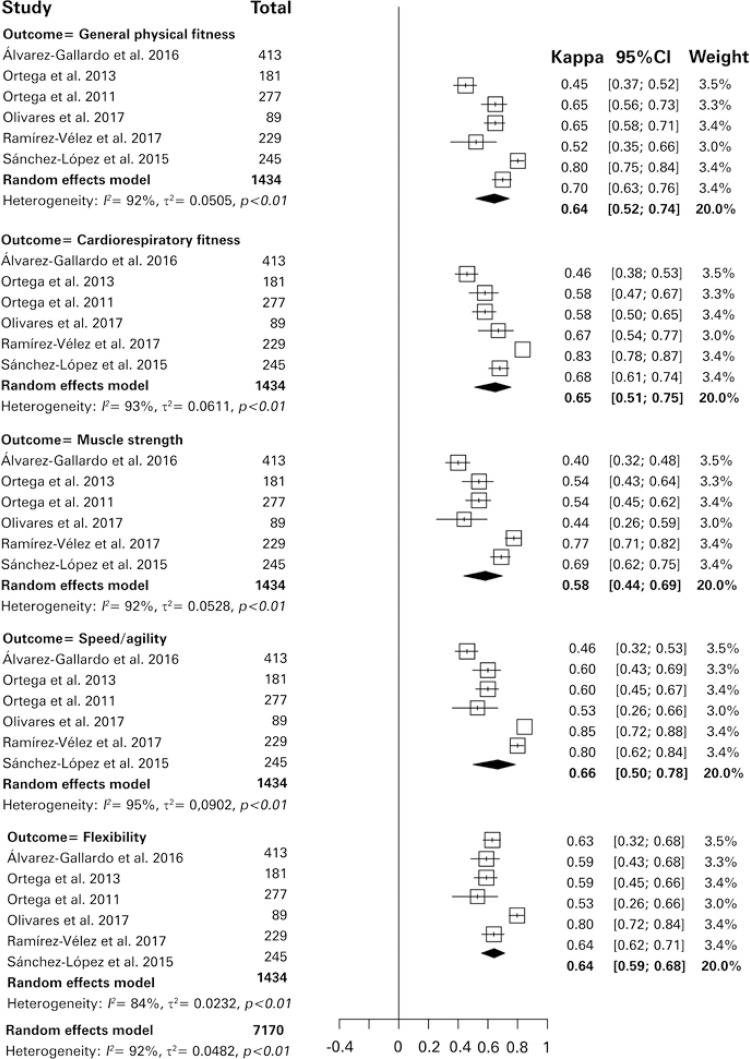
Comparative test-retest reliability of International Fitness Scale items among studies 95%CI: 95% confidence interval.

Kappa coefficients attributed to IFIS domains in selected studies were as follows: overall physical fitness - moderate, substantial and almost perfect agreement in two, four and two articles, respectively; cardiorespiratory fitness - moderate, substantial and almost perfect agreement in three articles, respectively; muscle strength - moderate, substantial, fair and almost perfect agreement in three, two, one and two articles, respectively; speed/agility - moderate, substantial and almost perfect agreement in four, one and three articles, respectively; flexibility – substantial, moderate and almost perfect agreement in three, three and two articles, respectively ( Figure 2 ).

### Sensitivity analysis

Lower Kappa coefficients attributed to the adult population compared to other subgroups in all domains suggest moderate agreement in that population ( Table 4 ). Risk of sampling bias across studies may significantly affect agreement in overall fitness (p<0.001), cardiorespiratory fitness (p<0.001), muscle strength (p=0.022) and flexibility (p<0.001) IFIS domains ( Table 5 ).

**Table 4:** Subgroup analysis

Population	Children (k=1)	Adolescents (k=2)	Adults (k=5)	Test for subgroup differences
IFIS Domains	Kappa (95%CI)	Kappa (95%CI)	Kappa (95%CI)	p value
Overall fitness	0.90 (0.98-0.99)	0.76 (0.60-0.85)	0.55 (0.33-0.72)	<0.001
I^2^	-	95.0%	90.5%	
Cardiorespiratory fitness	0.98 (0.97-0.98)	0.80 (0.59-0.91)	0.51 (0.39-0.62)	<0.001
I^2^	-	97.7%	70.5%	
Muscle strength	0.95 (0.93-0.96)	0.73 (0.53-0.85)	0.46 (0.32-0.59)	<0.001
I^2^	-	96.2%	75.3%	
Speed/agility	0.98 (0.97-0.98)	0.76 (0.62-0.86)	0.53 (0.38-0.65)	<0.001
I^2^	-	94.5%	79.0%	
Flexibility	0.93 (0.91-0.95)	0.73 (0.57-0.83)	0.62 (0.57-0.67)	<0.001
I^2^	-	94.3%	0.0%	

IFIS: International Fitness Scale; 95%CI: 95% confidence interval.

**Table 5:** Subgroup analysis

Risk of bias Q2_QAREL	Yes (k=2)	No (k=2)	Not clear (k=4)	Test for subgroup differences
IFIS domains	Kappa (95%CI)	Kappa (95%CI)	Kappa (95%CI)	p value
Overall physical fitness	0.67 (0.62-0.72)	0.97 (0.83-0.99)	0.63 (0.41-0.77)	<0.001
I^2^	8.1%	98.5%	94.8%	
Cardiorespiratory fitness	0.63 (0.52-0.72)	0.98 (0.96-0.98)	0.66 (0.43-0.81)	<0.001
I^2^	72.0%	65.1%	95.8%	
Muscle strength	0.62 (0.45-0.75)	0.94 (0.92-0.96)	0.56 (0.32-0.74)	0.022
I^2^	86.9%	51.1%	94.9%	
Speed/agility	0.71 (0.46-0.86)	0.95 (0.73-0.99)	0.64 (0.37-0.81)	0.097
I^2^	95.3%	98.3%	96.5%	
Flexibility	0.61 (0.56-0.66)	0.92 (0.88-0.94)	0.66 (0.52-0.76)	<0.001
I^2^	0.0%	57.6%	89.3%	

IFIS: The International Fitness Scale; Q2_QAREL: Quality Assessment of Reliability Studies checklist; 95%CI: 95% confidence interval.

More strict studies regarding risk of bias assessment as per Q2 had lower Kappa coefficients compared to other subgroups. As regards heterogeneity, meta-regression revealed that both subgroups (population and risk of bias as per Q2_QAREL) explained 85.99% of overall heterogeneity among studies (Tables 4 and 5). Summarized findings and GRADE quality classifications are presented in table 6 .

**Table 6:** Summarized findings

IFIS compared to test-retest for physical fitness measurement						
Population: children, adolescents, adults and women with fibromyalgia						
Context: IFIS application to measure physical fitness						
Intervention: IFIS						
Comparison: test-retest						

Outcomes	Potential absolute effects* (95%CI)		Relative effect (95%CI)	Number of participants (studies)	Certainty of evidence (GRADE)	Comments

	Reliability with test-retest	Reliability with IFIS				

Physical fitness as per IFIS Follow-up: 1 week to 2 weeks	-	Kappa 0.8 (0.56-0.92)	-	1,734 (7 observational studies)	⨁ ◯ ◯ ◯Very low^*^	-
Cardiorespiratory fitness as per IFIS Follow-up: 1 week to 2 weeks	-	Kappa 0.81 (0.59-0.92)	-	1,734 (7 observational studies)	⨁ ◯ ◯ ◯ Very low^*^	-
Muscle strength as per IFIS Follow-up: 1 week to 2 weeks	-	Kappa 0.73 (0.53-0.86)	-	1,734 (7 observational studies)	⨁ ◯ ◯ ◯ Very low^*^	-
Speed/agility as per IFIS Follow-up: 1 week to 2 weeks	-	Kappa: 0.79 (0.57-0.9)	-	1,734 (7 observational studies)	⨁ ◯ ◯ ◯ Very low^*^	-
Flexibility as per IFIS Follow-up: 1 week to 2 weeks	-	Kappa 0.74 (0.61-0.84)	-	1,734 (7 observational studies)	⨁ ◯ ◯ ◯ Very low^*^	-

* Reduction of two levels of evidence for reliability due to the unexplained substantial heterogeneity, and reduction of level of evidence for reliability due to the indirect evidence. A difference was observed in population profile in the studies.

IFIS: International Fitness Scale; 95%CI: 95% confidence interval.

⨁ Very low; ⨁⨁ Moderate; ⨁⨁⨁ High and ⨁⨁⨁⨁ Very high.

## DISCUSSION

Global organizations, such as the World Health Organization (WHO) and the American College of Sports Medicine (ACSM) currently recommend regular practice of moderate to vigorous physical activity for 150 minutes per week for overall physical fitness improvement.^( 18 , 19 )^

A retrospective cohort study following up on 122,007 patients revealed that cardiorespiratory fitness is inversely associated with long term mortality.^( 20 )^ Combined with findings of that study, a meta-analysis involving 2,525,827 adults revealed progressive decline in health parameters and increased obesity and related comorbidity rates as cardiorespiratory fitness decreases.^( 19 )^

Physical fitness is a health problem predictor and a modifiable indicator. It should therefore be assessed via gold-standard tests, such as cardiorespiratory fitness (ergospirometry),^( 21 )^ muscle strength (isokinetic test),^( 22 )^ speed/agility (20/40 m sprint test using photocell systems)^( 23 )^ and flexibility (inclinometer, goniometer, Leighton flexometer, fleximeter and imaging methods, like radiography and photogrammetry).^( 24 , 25 )^

However, application of aforementioned tests in scarce financial resource settings, or when specialized personnel is lacking, is not feasible and may preclude large scale studies.^( 26 )^ Hence the interest in alternative, user-friendly, low-cost tool development by public health organizations and researchers working in developing countries.

This is the first systematic review and meta-analysis investigating IFIS reliability – or consistency over time – based on test-retest, which is a significant aspect of any assessment tool. Low test-retest reliability tools are not able to detect true score changes over time.^( 9 )^

Overall, findings of this study revealed that test-retest reliability of IFIS domains determined using Kappa coefficients of agreement is valid for assessing overall physical fitness and related components (cardiorespiratory fitness, muscle strength, speed/agility and flexibility), given the low variability in reliability measures and moderate to substantial scores attributed to most domains.

In this study, steps were controlled via a systematic approach and strict protocol. Comprehensive search with no restrictions regarding study type, population, language, age, sex and date of publication was also conducted. Besides other advantages of questionnaires, IFIS has significant clinical applicability, once findings are associated with directly measured cardiorespiratory fitness and risk factors for cardiovascular disease, such as adiposity and metabolic syndrome, in different populations.^( 3 , 6 , 8 )^ Physical fitness assessment is also a critical indicator for ideal, personalized prescription of physical exercise.^( 7 )^

In spite of acceptable Kappa coefficient values, results of this meta-analysis involve potential risk of bias and overestimation. This heterogeneity was in part attributed to test-retest reliability dispersion across different populations. Some authors reported high test-retest reliability among measures in children, whereas others reported medium and low values in adolescents and adults, respectively. Low methodological quality (QAREL items Q4-Q7) may also have compromised reliability, as selected studies in this sample failed to satisfy these criteria.^( 11 )^ Also, the IFIS version used by De Moraes et al.,^( 17 )^has not been validated for the Brazilian population.

High heterogeneity among items detected in sensitivity analysis indicates that health status, age group, blinding of raters, test-retest time intervals, questionnaire application instructions and understanding by volunteers^( 7 , 3 )^ may impact study findings.

Therefore, interpretation and generalization of findings reported here must be done with caution, since this meta-analysis excluded grey literature and the few studies investigating IFIS reliability were of low methodological quality and involved high statistical heterogeneity according to grouped Kappa coefficients.

Finally, the fact that IFIS is available in nine languages must be emphasized. Should it be applied without previous adaptation and testing in samples with different characteristics from those accounted for in instrument construction and testing, cultural bias may occur. In order not to compromise findings of future Brazilian studies, application of the Portuguese version of IFIS and reference to Guidelines for Reporting Reliability and Agreement Studies (GRRAS)^( 27 )^ and QAREL checklist^( 12 )^ are recommended.

## CONCLUSION

Documentary *corpus* in this meta-analysis revealed high heterogeneity among studies, in spite of almost perfect agreement in 30% of items and appropriate item test-retest scores in most cases, which suggests moderate to substantial reliability according to Kappa coefficients.

Hence, further studies with low risk of bias and investigating instrument reliability and health status in different populations are needed to support the reliability of the self-reported International Fitness Scale questionnaire as an alternative tool for large scale physical fitness assessment or follow-up and an alternative ancillary test.
