# Supplementary material for: Reliability of questionnaire The International Fitness Scale: a systematic review and meta-analysis
Source: Einstein (Sao Paulo). 2020 Jul 23;18:eRW5232. doi: 10.31744/einstein_journal/2020RW5232 (PMC7394011; doi:10.31744/einstein_journal/2020RW5232)
Supplement: Translation

# Translation: Confiabilidade do questionário *The International Fitness Scale* : uma revisão sistemática e metanálise

**Authors:** Débora de Almeida Pereira, Jânio Luiz Correia, Nelson Carvas, Ricardo de Freitas-Dias

**Affiliations:** 1 Brasil Programa de Pós-Graduação em Hebiatria - Determinantes da Saúde na Adolescência, Universidade de Pernambuco, Camaragibe, PE, Brasil.; 2 Brasil Departamento de Fisioterapia, Universidade Pernambuco, Petrolina, PE, Brasil.; 3 Brasil Universidade Ibirapuera, São Paulo, SP, Brasil.

**Keywords:** The International Fitness Scale, IFIS, Aptidão física, Força muscular, Aptidão cardiorrespiratória, Autorrelato

## Abstract

**Objetivo:**

Realizar uma revisão sistemática da literatura e metanálise para verificar se o questionário *The International Fitness Scale* apresenta boa confiabilidade na avaliação da aptidão física geral e seus componentes.

**Métodos:**

A busca bibliográfica realizou-se nas bases de dados: PubMed^®^, BIREME, SciELO, EMBASE, SPORTDiscus, LILACS e Cochrane, a partir dos termos: *“The International Fitness Scale* ”, “ *International Fitness Scale* ” e “IFIS”. O processo de seleção e extração dos dados seguiram os critérios de elegibilidade: ser estudo de confiabilidade e/ou validade de instrumentos de medida do *The International Fitness Scale* ; ter o *The International Fitness Scale* como critério de referência (padrão-ouro); e ser artigo original. A qualidade dos estudos foi avaliada pelo *Assessment of Reliability Studies* . Para análise dos dados, utilizaram-se o coeficiente de concordância de Kappa, o teste Cochran e o I^2^de Higgins; para análise de sensibilidade, foi usado o modelo de retirada.

**Resultados:**

No total, sete artigos foram incluídos na análise. Os coeficientes de confiabilidade teste-reteste dos estudos variaram de 0,40 a 0,99, sendo a maioria representada por valores ≥0,60, indicando de moderada a substancial confiabilidade.

**Conclusão:**

Apesar dos indicadores de confiabilidade apresentarem um escore adequado para o teste-reteste, a heterogeneidade entre os estudos permaneceu elevada, necessitando de mais pesquisas com baixo risco de viés, para que o *The International Fitness Scale* autorrelatado seja considerado alternativa confiável.

## INTRODUÇÃO

A aptidão física é um dos preditores dos problemas de saúde e, quando em níveis satisfatórios, favorece a prevenção, a manutenção e a melhoria da capacidade funcional, além de reduzir o desenvolvimento de disfunções crônico-degenerativas, aumentando a qualidade de vida do indivíduo.^( 1 )^

Métodos de mensuração direta da aptidão física considerados padrão-ouro apresentam limitações, devido à necessidade de laboratórios, ao elevado custo de equipamentos, à equipe especializada e à difícil interpretação dos resultados.^( 2 , 3 )^Entretanto, os questionários são alternativas para estudos epidemiológicos, principalmente em países em desenvolvimento,^( 4 )^devido à facilidade de aplicação, ao baixo custo, à fidedignidade e à reprodutibilidade.^( 5 )^

Pesquisas multicêntricas relacionadas ao estilo de vida de adolescentes na Europa, desenvolveram o questionário *The International Fitness Scale* (IFIS) para avaliação autorrelatada da aptidão física geral e de seus componentes (capacidade cardiorrespiratória, força muscular, velocidade/agilidade e flexibilidade).^( 2 )^Ele foi originalmente validado no idioma inglês, para adolescentes de 12 a 17 anos.^( 2 )^Posteriormente, foi adaptado e traduzido para nove idiomas (alemão, alemão austríaco, grego, flamengo, francês, húngaro, italiano, espanhol e sueco)^( 2 )^e validado em diferentes populações (crianças, jovens e adultos de ambos os sexos).^( 3 , 6 - 8 )^Seus resultados apresentaram associação com fatores de risco de doenças cardiovasculares e síndrome metabólica.^( 3 , 6 , 8 )^

Apesar do IFIS ser utilizado em várias pesquisas internacionais, estudos que buscam estimar a aptidão física, identificar fatores de risco associados, analisar sua relação com diferentes desfechos e avaliar a efetividade de programas de treinamento necessitam que seus instrumentos apresentem propriedades psicométricas acuradas, capazes de reproduzir determinado resultado de forma consistente no tempo e no espaço, ou a partir de observadores diferentes (confiabilidade).^( 9 )^

Considerando a importância de mensurar a aptidão física e o interesse crescente nesta área, utilizando instrumentos de boa confiabilidade e fácil aplicação, optou-se realizar uma revisão sistemática e metanálise da literatura, para verificar se o IFIS é uma ferramenta que apresenta boa confiabilidade para avaliar a aptidão física geral e seus componentes.

## MÉTODOS

### Protocolo e registro

Esta revisão sistemática seguiu as recomendações dos *Reporting Items for Systematic Reviews and Meta-Analyses* (PRISMA), e o protocolo de revisão foi registrado no *International Prospective Register of Systematic Reviews* (PROSPERO) sob o número CRD42018117472.

### Estratégia de busca

A busca bibliográfica foi realizada até o mês de setembro de 2019 nas bases de dados MEDLINE via PubMed^®^, BIREME, *Scientific Electronic Library Online* (SciELO), EMBASE, SPORTDiscu *s* , LILACS e Cochrane Central, sem restrição de tipo de estudo, população, idioma, idade e sexo do participante, e data de publicação. Para identificação dos estudos, foram realizadas buscas utilizando descritores controlados *“Physical Fitness”* e *“Self-report”,* e não controlados *:* “ *The International Fitness Scale* ”; “ *International Fitness Scale* ”; “IFIS”. Para combinar os termos, utilizou-se o operador booleano ( *OR* ) e, para exibir somente artigos que tinham os termos no título e resumo, utilizou-se a chave [TIAB] ( Tabela 1 ).

**Tabela 1:** Estratégia de busca

Nas bases de dados PubMed^®^, BIREME, SciELO, EMBASE, SPORTDiscus, LILACS e Cochrane: *The International Fitness Scale* [TIAB] *OR* [TIAB] *International Fitness Scale* [TIAB] OR IFIS [TIAB]
Na EMBASE, como não é permitido utilizar a chave [TIAB]: *The International Fitness Scale OR International Fitness Scale* [TIAB] *OR* IFIS.

### Seleção de estudos

Para a seleção dos estudos, foi desenvolvido um formulário de avaliação, com base nos critérios de inclusão e exclusão, sendo realizada sua calibração antes da triagem. Os critérios de inclusão foram ser estudo de confiabilidade e/ou validade do instrumento de medida do IFIS; ser artigo original de pesquisa desenvolvida com seres humanos; e ter sido publicado em periódico indexado nas bases utilizadas. Foram excluídos artigos de revisão. Para garantir a seleção e a avaliação independente entre os revisores, foi utilizado o *Mendeley Reference Manager Software* (https://www.mendeley.com/).

Após a exclusão dos estudos duplicados, dois revisores, de forma cega e independente, realizaram a seleção dos estudos em duas etapas: triagem de títulos e resumos, e leitura na íntegra. Na primeira etapa, todos os títulos e resumos encontrados foram avaliados, a partir dos critérios de elegibilidade predefinidos para identificação de estudos relevantes. Caso pelo menos um dos avaliadores incluísse um estudo durante esta etapa, o mesmo era incluído na etapa subsequente. De posse dos artigos selecionados, seguiu-se à leitura completa dos textos, sendo utilizado, pelos avaliadores, um formulário de avaliação, com base nos critérios de elegibilidade.

Objetivando identificar algum estudo relevante que não tinha sido encontrado na busca eletrônica, efetuou-se a busca cruzada, a partir da análise das referências dos artigos selecionados para a leitura na íntegra.

### Extração dos dados

A extração de dados foi conduzida de acordo com a orientação do Manual Cochrane de Revisões Sistemáticas de Intervenções.^( 10 )^Para todos os estudos que atendiam aos critérios de elegibilidade, foram tabuladas em uma planilha eletrônica do software Microsoft Excel (Microsoft Corporation, WA, EUA) as seguintes informações: primeiro autor do estudo, título e ano de publicação; tipo de estudo; dados descritivos (número da amostra total e por sexo, faixa etária, país onde foi realizado o estudo, procedimento de seleção da amostra) e dados de confiabilidade (valores Kappa e IC95%).

Dois avaliadores extraíram, de forma independente, os dados descritivos e o desfecho de cada artigo incluído. Para avaliar a qualidade geral da evidência, utilizou-se o Sistema GRADE.^( 11 )^Todas as discrepâncias que não puderam ser resolvidas por consenso entre os dois avaliadores foram julgadas por um terceiro avaliador. Antes do processo de extração, os avaliadores passaram por um treinamento de calibração, para garantir a consistência entre os revisores e o refinamento da planilha de extração dos dados.

### Avaliação da qualidade metodológica dos estudos: risco de viés

A qualidade dos estudos incluídos foi avaliada pela ferramenta *Quality Appraisal of Reliability Studies* (QAREL), que contém 11 itens divididos nos seguintes domínios: itens 1 e 2 levam em consideração o viés de amostragem, os participantes e a representatividade do avaliador; itens 3 a 7 incluem o cegamento do avaliador; o item 8 pergunta se a sequência do teste variou; o item 9 leva em conta se o intervalo de tempo entre as medidas foi suficiente; o item 10 analisa se o teste foi realizado e interpretado corretamente; e o item 11 leva em consideração a adequação das análises estatísticas. Cada item pode ser respondido com “sim”, “não”, “impreciso” e “não aplicável” (itens 3, 4, 5, 6 e 8), sendo uma resposta “sim” sugestiva de um recurso de boa qualidade do estudo, e uma resposta “não” de um recurso de má qualidade.^( 12 )^

Quando ocorreu discordância em qualquer etapa deste trabalho, os autores discutiram suas razões, e a decisão final foi feita por consenso, conforme recomendações do *Handbook* para revisões sistemáticas da colaboração Cochrane.^( 10 )^Na ausência de consenso, a arbitragem de um terceiro autor era solicitada, sendo julgadas e resolvidas as razões para a exclusão dos artigos.

### Análise dos dados

Para a confiabilidade, utilizou-se o coeficiente de concordância de Kappa, e o tamanho amostral foi usado para calcular o Kappa agrupado. O modelo de efeitos aleatórios foi escolhido sobre o de efeito fixo, devido à variação do nível de aptidão física entre os indivíduos, podendo ser explicada pelo nível de atividade física durante a infância e a adolescência, o qual influencia na vida adulta.^( 13 )^Para interpretação dos coeficientes de concordância de Kappa, foram utilizadas as seguintes categorias: nenhum <0,00; leve, de 0,00 a 0,20; pequena, de 0,21 a 0,40; moderada, de 0,41 a 0,60; substancial, de 0,61 a 0,80; e quase perfeita, de 0,81 a 1,00.^( 14 )^

A heterogeneidade estatística foi avaliada por meio do teste Cochran Q (adotando o nível de significância p<0,10), e a inconsistência foi avaliada pelo I^2^de Higgins,^( 15 )^com valores de ≤40% indicando baixa heterogeneidade; 30% a 60%, heterogeneidade moderada; >50% a 90%, heterogeneidade substancial; e >75% a 100%, heterogeneidade considerável.^( 10 )^Quando I^2^>50% e *tau squared* (𝛕^2^) >1, acompanhado pela significância estatística (p<0,10), considerou-se que havia heterogeneidade significativa, e a razão disso foi investigada. Todas as análises foram realizadas no software estatístico programa R versão 3.5.1 utilizando o pacote meta.

### Análise de sensibilidade

Para explicar a heterogeneidade dos estudos, realizou-se a análise de subgrupos, dividindo os efeitos pela população estudada e pelo risco de viés da representação da amostra, seguido do cálculo da metaregressão.

## RESULTADOS

Foram encontrados 1.999 artigos nas bases de dados, dos quais 871 foram eliminados por estarem duplicados. Para a triagem do título/resumo, foram analisados 1.128 artigos e, para texto completo, 23 artigos, obtendo 99,2% de concordância entre os avaliadores. Dentre eles, sete artigos científicos foram incluídos na análise quantitativa e narrativa desta metanálise, por terem analisado e apresentado os dados de confiabilidade ( Figura 1 ).

**Figura 1:** Fluxograma da seleção dos estudos IFIS: *The International Fitness Scale* .

### Características do estudo

Para a síntese narrativa e quantitativa desta metanálise, foram incluídos sete estudos.^( 2 , 3 , 6 , 7 , 8 , 16 , 17 )^O período de publicação desses artigos selecionados variou entre 2011 e 2019, e o tamanho amostral entre 89 e 413. No geral, cinco estudos^( 2 , 3 , 6 , 8 , 16 )^incluíram participantes de ambos os sexos, sendo o sexo masculino predominante em três estudos,^( 3 , 8 , 16 )^um estudo utilizou amostra exclusiva de mulheres^( 7 )^e um estudo que não informou a distribuição do sexo da amostra.^( 17 )^O processo de recrutamento das amostras foi principalmente por amostragem aleatória,^( 2 , 6 , 7 , 8 , 16 )^e dois deles realizaram amostragem por conveniência.^( 3 , 17 )^Relativo à idade dos participantes, foi verificada grande heterogeneidade entre os estudos, no qual sua média variou de 3 a 65 anos. Quanto ao tipo do estudo, cinco eram observacionais do tipo confiabilidade teste-reteste,^( 2 , 3 , 6 , 16 , 17 )^um ensaio randomizado em *cluster,* ^( 8 )^e um estudo transversal^( 7 )^( Tabela 2 ).

**Tabela 2:** Síntese e características dos resultados dos estudos de confiabilidade do instrumento *The International Fitness Scale* para a medida da aptidão física

Autor	Tipo de estudo	Tamanho amostral	Características dos participantes	Intervalo entre as aplicações	Aplicação do IFIS
Ortega et al.^(2)^	Observacional do tipo confiabilidade teste-reteste	n=277	Sexo: feminino (51%) e masculino (49%). Faixa etária: 12,5 a 17,5 anos. Países: Grécia, Alemanha, Bélgica, França, Hungria, Áustria, Itália, Suécia e Espanha. Condição de saúde: livre de qualquer infecção aguda ou doença duradoura <1 semana antes da inclusão no estudo	2 semanas	Não relatou
Ramírez-Velez et al.^(3)^	Observacional do tipo confiabilidade teste-reteste	n=229	Sexo: feminino (45,85%) e masculino (54,15%). Faixa etária: 9 a 17,9 anos. País: Colômbia. Condição de saúde: não possuir diagnóstico clínico de doença cardiovascular e/ou *diabetes mellitus* tipos 1 ou 2, não estar grávida, e consumir álcool ou outras drogas	1 semana	Não relatou
Ortega et al.^(6)^	Observacional do tipo confiabilidade teste-reteste	n=181	Sexo: feminino (73,48%) e masculino (27,52%). Faixa etária: 18 a 43 anos. País: Espanha	2 semanas	Não relatou
Álvarez-Gallardo et al.^(7)^	Estudo transversal	n=413	Sexo: feminino. Faixa etária: 37 a 65 anos. País: Espanha. Condição de saúde: portadoras de fibromialgia	1 semana	Não relatou
Sánchez-López et al.^(8)^	Ensaio randomizado em *cluster*	n=245	Sexo: feminino (54%) e masculino (46%). Faixa etária: 9 a 12 anos. País: Espanha	2 semanas	Não relatou
Olivares et al.^(16)^	Observacional do tipo teste-reteste	n=89	Sexo: feminino (37,8%) e masculino (62,2%). Faixa etária: 12 a 18 anos. País: Chile	2 semanas	Realizado durante a aula de educação física Os aplicadores eram licenciados em Pedagogia em Educação Física Os aplicadores foram treinados previamente
De Moraes et al.^(17)^	Observacional do tipo confiabilidade teste-reteste	n=190 crianças e n=110 adolescentes	A distribuição do sexo dos adolescentes participantes não foi informada pelos autores. Crianças com faixa etária de 3 a 10 anos (média 6,7±2,1 anos) e adolescentes com faixa etária de 11 a 17 anos (14,6±1,8 anos). País: Brasil	15 dias	A coleta dos dados foi realizada em 5 visitas: (1) Explicação do projeto e entrega do TCLE aos pais; (2) Entrega do questionário para autorrelato; (3) coleta do questionário preenchido; (4) segunda aplicação; (5) coleta do questionário preenchido

IFIS: *International Fitness Scale* ; TCLE: Termo de Consentimento Livre Esclarescido.

Os resultados dos estudos apresentaram medidas de confiabilidade mediante o procedimento teste-reteste, estimado pelo coeficiente de concordância de Kappa. O intervalo de tempo entre as aplicações variou de 1 a 2 semanas, sendo 2 semanas o período mais utilizado,^( 2 , 6 , 8 , 16 , 17 )^porém, dois estudos utilizaram 1 semana de intervalo.^( 3 , 7 )^

### Risco de viés

A concordância entre os dois avaliadores quanto ao risco de viés foi de 94,8% (4 discordâncias em 77 itens avaliados). No geral, todos os estudos^( 2 , 3 , 6 , 7 , 8 , 16 , 17 )^incluíram participantes representativos da população, no qual os autores consideravam que os resultados fossem aplicados (item Q2 do QAREL) e também informaram o intervalo de medida da variável avaliada (item Q9 do QAREL).

Relativo às fontes primárias de viés, nenhum estudo relatou o cegamento dos avaliadores para as conclusões de outros avaliadores, para suas próprias descobertas anteriores do teste em avaliação, para os resultados do padrão de referência aceito para a variável avaliada, para informações clínicas, para outras avaliações de avaliadores e ordem da avaliação. Dois estudos^( 2 , 6 )^realizaram o teste com avaliadores representativos daqueles a quem os autores pretendiam que os resultados fossem aplicados. Por fim, todos os estudos analisados, aplicaram e interpretaram corretamente o teste, bem como realizaram a análise estatística correta ( Tabela 3 ).

**Tabela 3:** Avaliação da qualidade metodológica utilizando o *Quality Assessment of Reliability Studies checklist*

Estudo	Q1	Q2	Q3	Q4	Q5	Q6	Q7	Q8	Q9	Q10	Q11
Ortega et al.^(2)^	S	S	NC	NC	NC	NC	NC	NC	S	S	S
Ramírez-Vélez et al.^(3)^	S	NC	NC	NC	NC	NC	NC	NC	S	S	S
Ortega et al.^(6)^	S	NC	NC	NC	NC	NC	NC	NC	S	S	S
Álvarez-Gallardo et al.^(7)^	S	NC	NC	NC	NC	NC	NC	NC	S	S	S
Sánchez-López et al.^(8)^	S	S	NC	NC	NC	NC	NC	NC	S	S	S
Olivares et al.^(16)^	S	NC	NC	NC	NC	NC	NC	NC	S	S	S
De Moraes et al.^(17^	S	NC	NC	NC	NC	NC	NC	NC	S	S	S

Q1: O teste foi avaliado em uma amostra de sujeitos que foram representativos daqueles a quem os autores pretendiam que os resultados fossem aplicados?; Q2: O teste foi realizado em amostras representativas daqueles a que os autores pretendiam que os resultados fossem aplicados?; Q3: Os avaliadores foram cegados para as conclusões de outros avaliadores durante o estudo?; Q4: Os avaliadores foram cegados para suas próprias descobertas anteriores do teste em avaliação?; Q5: Os avaliadores foram cegados para os resultados do padrão de referência aceito para a variável que está sendo avaliada?; Q6: Os avaliadores foram cegados para informações clínicas que não se destinavam fazer parte do procedimento de teste ou desenho do estudo?; Q7: Os avaliadores foram cegados para sugestões adicionais que não fazem parte do teste?; Q8: A ordem do exame foi variada?; Q9: O intervalo de tempo entre as medidas repetidas foi compatível com a estabilidade (ou estabilidade teórica) da variável que está sendo medida?; Q10: O teste foi aplicado corretamente e interpretado adequadamente?; Q11: Foram utilizadas medidas estatísticas apropriadas?; S: sim; NC: não claro.

### Síntese dos resultados de confiabilidade

No geral, o coeficiente total de confiabilidade teste-reteste a partir do Kappa variou de 0,73 a 0,81, representando nível de substancial a quase perfeito. De acordo com todos os itens dos estudos, houve variação de 0,40 a 0,99 (pequeno a quase perfeito), sendo mais de 50% (26 de 40 itens) com valores ≥0,60, que é considerado de moderado a substancial nível de confiabilidade, e 30% (12 de 40 itens) representados por nível quase perfeito, segundo os parâmetros descritos por Landis et al.^( 14 )^

Referente ao coeficiente de concordância de Kappa dos domínios avaliados pelo IFIS, dos artigos analisados, dois apresentaram nível moderado, quatro substancial e dois quase perfeito para a aptidão física geral; três apresentaram nível moderado, dois substancial e três quase perfeito para capacidade cardiorrespiratória; três apresentaram nível moderado, dois substancial, um pequeno e dois quase perfeito para força muscular; quatro apresentaram nível moderado, um substancial e três quase perfeito para velocidade/agilidade; e três apresentaram nível substancial, três moderado e dois quase perfeito para flexibilidade ( Figura 2 ).

**Figura 2:** Comparação da confiabilidade teste-reteste dos itens do *The International Fitness Scale* entre os estudos IC95%: intervalo de confiança 95%.

### Análise de sensibilidade

Pode-se observar que a população adulta apresenta valores de coeficiente de concordância de Kappa mais baixos que os outros subgrupos em todos os domínios, sugerindo concordância moderada para esta população ( Tabela 4 ). O risco de viés na representatividade da amostra entre os estudos pode afetar significativamente a concordância do IFIS nos domínios aptidão geral (p<0,001), capacidade cardiorrespiratória (p<0,001), força muscular (p=0,022) e flexibilidade (p<0,001) ( Tabela 5 ).

**Tabela 4:** Análise de subgrupos

População	Crianças (k=1)	Adolescentes (k=2)	Adultos (k=5)	Teste para diferença de subgrupos
Domínios do IFIS	Kappa (IC95%)	Kappa (IC95%)	Kappa (IC95%)	Valor de p
Aptidão geral	0,90 (0,98-0,99)	0,76 (0,60-0,85)	0,55 (0,33-0,72)	<0,001
I^2^	-	95,0%	90,5%	
Capacidade cardiorrespiratória	0,98 (0,97-0,98)	0,80 (0,59-0,91)	0,51 (0,39-0,62)	<0,001
I^2^	-	97,7%	70,5%	
Força muscular	0,95 (0,93-0,96)	0,73 (0,53-0,85)	0,46 (0,32-0,59)	<0,001
I^2^	-	96,2%	75,3%	
Velocidade/agilidade	0,98 (0,97-0,98)	0,76 (0,62-0,86)	0,53 (0,38-0,65)	<0,001
I^2^	-	94,5%	79,0%	
Flexibilidade	0,93 (0,91-0,95)	0,73 (0,57-0,83)	0,62 (0,57-0,67)	<0,001
I^2^	-	94,3%	0,0%	

IFIS: *The International Fitness Scale* ; IC95%: intervalo de confiança 95%.

**Tabela 5:** Análise de subgrupos

Risco de viés Q2_QAREL	Sim (k=2)	Não (k=2)	Não está claro (k=4)	Teste para diferença de subgrupos
Domínios do IFIS	Kappa (IC95%)	Kappa (IC95%)	Kappa (IC95%)	Valor de p
Aptidão geral	0,67 (0,62-0,72)	0,97 (0,83-0,99)	0,63 (0,41-0,77)	<0,001
I^2^	8,1%	98,5%	94,8%	
Capacidade cardiorrespiratória	0,63 (0,52-0,72)	0,98 (0,96-0,98)	0,66 (0,43-0,81)	<0,001
I^2^	72,0%	65,1%	95,8%	
Força muscular	0,62 (0,45-0,75)	0,94 (0,92-0,96)	0,56 (0,32-0,74)	0,022
I^2^	86,9%	51,1%	94,9%	
Velocidade/agilidade	0,71 (0,46-0,86)	0,95 (0,73-0,99)	0,64 (0,37-0,81)	0,097
I^2^	95,3%	98,3%	96,5%	
Flexibilidade	0,61 (0,56-0,66)	0,92 (0,88-0,94)	0,66 (0,52-0,76)	<0,001
I^2^	0,0%	57,6%	89,3%	

IFIS: *The International Fitness Scale* ; Q2_QAREL: *Quality Assessment of Reliability Studies* checklist; IC95%: intervalo de confiança 95%.

Os estudos mais rigorosos referentes à avaliação do risco de viés Q2 apresentaram valores de coeficiente de concordância de Kappa mais baixos que os outros subgrupos. Quanto à heterogeneidade, a metarregressão revelou que ambos os subgrupos (população e risco de viés na Q2_QAREL) explicam 85,99% da heterogeneidade total entre os estudos (Tabelas 4 e 5). A tabela 6 fornece o resumo dos resultados e a classificações de qualidade GRADE.

**Tabela 6:** Sumário de resultados

O IFIS comparado a teste-reteste para medir o nível de aptidão física						
**População:** crianças, adolescentes, adultos e mulheres portadoras de fibromialgia						
**Contexto:** aplicação do IFIS para medir o nível de aptidão física						
**Intervenção:** o IFIS						
**Comparação:** teste-reteste						

Desfechos	Efeitos absolutos potenciais^*^(IC95%)		Efeito relativo (IC95%)	Número de participantes (estudos)	*Certainty of the evidence* (GRADE)	Comentários

	Confiabilidade com teste-reteste	Confiabilidade com o IFIS				

Aptidão física avaliada com IFIS Seguimento: variação 1 semana para 2 semanas	-	Kappa 0,8 (0,56-0,92)	-	1.734 (7 estudos observacionais)	⨁ ◯ ◯ ◯ Muito baixa^*^	-
Capacidade cardiorrespiratória avaliada com IFIS Seguimento: variação 1 semana para 2 semanas	-	Kappa 0,81 (0,59-0,92)	-	1.734 (7 estudos observacionais)	⨁ ◯ ◯ ◯ Muito baixa^*^	-
Força muscular avaliada com IFIS Seguimento: variação 1 semana para 1 semanas	-	Kappa 0,73 (0,53-0,86)	-	1.734 (7 estudos observacionais)	⨁ ◯ ◯ ◯ Muito baixa^*^	-
Velocidade/agilidade avaliada com IFIS Seguimento: variação 1 semana para 2 semanas	-	Kappa 0,79 (0,57-0,9)	-	1.734 (7 estudos observacionais)	⨁ ◯ ◯ ◯ Muito baixa^*^	-
Flexibilidade avaliada com: IFIS Seguimento: variação 1 semana para 2 semanas	-	Kappa 0,74 (0,61-0,84)	-	1.734 (7 estudos observacionais)	⨁ ◯ ◯ ◯ Muito baixa^*^	-

* Redução de dois níveis na certeza da evidência devido a heterogeneidade substancial não explicada e redução de um nível na certeza da evidência devido a evidência indireta. Houve diferença no perfil populacional entre os estudos.

IFIS: *The International Fitness Scale* ; IC95%: intervalo de confiança de 95%.

⨁ Muito baixa; ⨁⨁ Moderada; ⨁⨁⨁ Alta e ⨁⨁⨁⨁ Muito alta.

## DISCUSSÃO

Atualmente, as instituições mundiais, como a Organização Mundial da Saúde (OMS) e *American College of Sports Medicine* (ACSM), recomendam a prática regular de 150 minutos por semana de atividade física em intensidade moderada a vigorosa, acarretando melhores níveis de aptidão física geral.^( 18 , 19 )^

Um estudo de coorte retrospectivo que acompanhou 122.007 pacientes verificou que a capacidade cardiorrespiratória é inversamente associada à mortalidade a longo prazo.^( 20 )^Associado a este estudo, os resultados de uma metanálise, que avaliou 2.525.827 adultos, verificaram que conforme ocorre redução da capacidade cardiorrespiratória, observam-se também declínio progressivo dos parâmetros gerais de saúde e aumento da obesidade e suas comorbidades.^( 19 )^

Sabendo que a aptidão física é um dos preditores de problemas de saúde e indicador modificável, recomenda-se sua avaliação por meio de testes considerados padrão-ouro, como capacidade cardiorrespiratória (ergoespirometria);^( 21 )^força muscular (teste isocinético);^( 22 )^velocidade/agilidade (teste de 20/40m utilizando o sistema de fotocélulas)^( 23 )^e flexibilidade (inclinômetro, goniômetro, flexômetro de Leighton e flexímetro, juntamente das análises de imagens, como radiografia e fotogrametria).^( 24 , 25 )^

Porém, a aplicação dos testes supracitados em locais que dispõem de recurso financeiro escasso e *deficit* de profissionais especializados inviabiliza estudos em larga escala.^( 26 )^Portanto, é de interesse das organizações de saúde pública e pesquisadores de países subdesenvolvidos e em desenvolvimento buscar instrumentos alternativos, de fácil acesso e baixo custo.

Esta é a primeira revisão sistemática e metanálise a avaliar a confiabilidade do IFIS, que significa a consistência de medição ao longo do tempo, a partir do teste-reteste, representando um aspecto importante de instrumentos que propõem avaliar algo. Se um instrumento tem baixa confiabilidade teste-reteste, indica que não consegue distinguir mudanças reais nas pontuações ao longo do tempo.^( 9 )^

De forma abrangente, os resultados mostraram que a confiabilidade dos domínios avaliados pelo IFIS mediante o procedimento teste-reteste, estimado pelo coeficiente de concordância de Kappa, é considerada válida para avaliar a aptidão física geral e seus componentes (capacidade cardiorrespiratória, força muscular, velocidade/agilidade e flexibilidade), principalmente por apresentar pequena variabilidade na medida de confiabilidade e, pelos domínios apresentarem, no geral, escores de moderado a substancial.

Neste estudo, foi seguida uma abordagem sistemática, utilizando protocolo rigoroso, que controlou suas etapas, além de ter sido realizada pesquisa abrangente, sem restrição para o tipo de estudo, população, idioma, idade, sexo e data de publicação.

Além das vantagens dos questionários, o IFIS apresenta importante papel na aplicabilidade clínica, pois seus resultados são associados à capacidade cardiorrespiratória medida diretamente e aos fatores de risco de doenças cardiovasculares, como adiposidade e síndrome metabólica, em diferentes populações.^( 3 , 6 , 8 )^Ademais, a avaliação da aptidão física é um indicador fundamental para individualizar a prescrição ideal de exercícios físicos.^( 7 )^

Apesar dos valores aceitáveis do coeficiente de concordância de Kappa, há possibilidade de viés e superestimação nos resultados da metanálise. Parte dessa heterogeneidade foi atribuída à dispersão da confiabilidade teste-reteste entre as diferentes populações. Enquanto alguns autores encontraram confiabilidade teste-reteste elevada entre as medidas para as crianças, outros mostraram valores médios para adolescentes e baixos para os adultos. Outro fator que pode comprometer é a baixa qualidade metodológica referente às questões Q4-Q7 do QAREL, visto que nenhum estudo cumpriu estes critérios,^( 11 )^e a falta de padronização do estudo de De Moraes et al.,^( 17 )^que utilizou uma versão do IFIS não validada para a população brasileira.

Seguida da análise da sensibilidade, os itens apresentaram elevada heterogeneidade, indicando que condição de saúde, faixa etária, cegamento dos avaliadores, intervalo de tempo entre o teste-reteste, instrução da aplicação do questionário e entendimento dos voluntários^( 7 , 3 )^podem influenciar no efeito entre os estudos.

Portanto, deve-se ter cautela na interpretação e na generalização destes resultados, já que esta metanálise não realizou a busca em literatura cinzenta, e os poucos estudos que avaliaram a confiabilidade do IFIS são de baixa qualidade e elevada heterogeneidade estatística, quando realizado o agrupamento dos coeficientes de concordância.

Por fim, é importante destacar que o IFIS está disponível em nove idiomas e, caso ele seja aplicado sem adaptação e testagem prévias em amostras com características distintas das quais eles foram desenvolvidos e avaliados, existe a possibilidade de ocorrência de viés cultural. Para evitar o comprometimento dos resultados de estudos futuros realizados no Brasil, recomendamos a utilização dos *Guidelines for Reporting Reliability and Agreement Studies* (GRRAS),^( 27 )^do *check-list* QAREL^( 12 )^para a avaliação da qualidade metodológica e da versão em português brasileiro do instrumento IFIS.

## CONCLUSÃO

Conclui-se com o *corpus* documental desta metanálise, que, apesar de a maioria dos itens apresentar escore adequado para o teste-reteste, indicando de moderada a substancial confiabilidade para o coeficiente de concordância Kappa, além de 30% dos itens apresentarem nível quase perfeito, a heterogeneidade entre os estudos permaneceu elevada.

Portanto, necessita-se de mais pesquisas, com baixo risco de viés, que testem a confiabilidade em diferentes populações e condições de saúde, para que o questionário autorrelatado *The International Fitness Scale* seja considerado alternativa confiável, podendo ser utilizado em larga escala para avaliação ou acompanhamento da aptidão física e fazer parte de exames complementares.
